# Proinflammatory profile in the skin of Parkinson’s disease patients with and without pain

**DOI:** 10.1371/journal.pone.0276564

**Published:** 2022-10-27

**Authors:** Joana Lama, Elena Salabasidou, Jens Volkmann, Anastasia Kuzkina, Susan Duty, Nurcan Üçeyler

**Affiliations:** 1 King’s College London, Institute of Psychiatry, Psychology and Neuroscience, Wolfson Centre for Age-Related Diseases, London, United Kingdom; 2 Department of Neurology, University of Würzburg, Würzburg, Germany; UCSI: UCSI University, MALAYSIA

## Abstract

**Background:**

Pain is a common non-motor symptom of Parkinson`s disease (PD), however, its pathomechanism remains elusive.

**Objective:**

We aimed to investigate the local gene expression of selected proinflammatory mediators in patients with PD and correlated our data with patients`pain phenotype.

**Methods:**

We recruited 30 patients with PD and 30 healthy controls. Pain intensity of patients was assessed using the Numeric Rating Scale (NRS) and patients were stratified into PD pain (NRS≥4) and PD No Pain (NRS<4) subgroups. Skin punch biopsies were immunoassayed for protein-gene product 9.5 as a pan-neuronal marker and intraepidermal nerve fiber density (IEFND). Quantitative real-time polymerase chain reaction (qRT-PCR) analysis was performed to assess the gene expression of inflammatory mediators in the skin compared to controls.

**Results:**

Patients with PD had lower distal IENFD compared to healthy controls. In skin samples, IL-2 (p<0.001) and TNF-α (p<0.01) were expressed higher in PD patients compared to controls. IL-1β (p<0.05) was expressed higher in the PD pain group compared to healthy controls. PD patients with pain receiving analgesics had a lower expression of TNF-α (p<0.05) in the skin compared to those not receiving treatment.

**Conclusions:**

Our data suggest the occurrence of a local, peripheral inflammatory response in the skin in PD, but do not support this being a relevant factor contributing to pain in PD.

## Introduction

The prevalence of Parkinson’s disease (PD) is increasing together with the associated personal, societal, and economic costs [1]. Key hallmarks of PD are the degeneration of dopaminergic neurons in the substantia nigra pars compacta in the midbrain and the accumulation of abnormal cerebral α-synuclein aggregates in Lewy bodies [2]. Clinically, PD manifests as bradykinesia, rigidity, and tremor as well as a variety of non-motor symptoms (NMS) involving depression, constipation, anxiety, sleep disorders, and pain [3]. Pain is one of the most debilitating NMS in PD substantially impairing health-related quality of life. The prevalence of pain in PD reaches 40–85% [4], however, specific analgesic treatment options are limited [5–7]. A better understanding of the underlying mechanisms of pain in PD will assist establishing novel and more effective therapeutics.

Several studies have reported that PD patients experience different types of pain in various parts of the body, however, a consensus on the classification of pain in PD is missing [8–13]. Ford’s classification of pain is currently the most commonly used one and subcategorizes the different types of pain in PD into musculoskeletal, dystonic, central, and radicular, the latter two subclassified as neuropathic [14]. Of the two forms of neuropathic pain, central pain in PD is assumed to be a direct consequence of the disease itself, and not an effect of the motor symptoms [14]. Central pain prevalence varies from 4–10% [13]. Radicular pain pathophysiology involves irritation or inflammation of the nerve roots [15] and the prevalence of this type of pain in PD varies from 14–35% [13]. Development and persistence of neuropathic pain has been associated with inflammation, as thoroughly reviewed elsewhere [16]. However, whether this applies to neuropathic pain in PD remains to be elucidated.

A number of studies have reported denervation in skin punch biopsies of PD patients compared to healthy controls [17–21]. One study reported elevated tactile and thermal thresholds and showed a reduction of epidermal nerve fibers and Meissner corpuscles [21]. In other studies, α-synuclein deposits were found in peripheral nerves as potential mediator of cutaneous denervation [22, 23].

In several studies, elevated systemic levels of proinflammatory cytokines were reported in PD patients [24]. Given that proinflammatory cytokines also have algesic effects [25–27], the question arises whether a local proinflammatory profile may have an impact on the development and/or persistence of pain in PD. Li et al. observed elevated systemic interleukin-1β (IL-1β) levels in a PD cohort experiencing pain compared to a healthy control group which may provide evidence for an inflammatory component in the pathophysiology of pain in PD [28].

In the present study, we explored the cutaneous gene expression of selected proinflammatory mediators in patients with PD and correlated our data with patients`pain phenotype. We hypothesized that PD patients reporting pain have a local proinflammatory profile compared to PD patients without pain.

## Materials and methods

### Patients and controls

PD patients and healthy controls were recruited at the Department of Neurology, University of Würzburg, Germany between December 2020 and August 2021. Thirty patients diagnosed with idiopathic PD fulfilling the clinical criteria according to the Movement Disorder Society with a median duration of 10.4 years (range 0–22) of PD were included in the study [29]. The median age of the patients was 67.8 years (range 54–81). The ratio of female to male was 3:10. Two individual control groups of healthy volunteers were used in our study: the first for RNA analysis (Control I), the second for skin innervation (Control II). Demographic data of the patients and controls are summarized in Table 1. Patients and controls provided oral and written informed consent to participate. All experiments were approved by the Medical School Ethics Committee of the University of Würzburg (#289/20).

**Table 1:** Demographic data of study cohort.

Characteristics	PD	PD Pain	PD No Pain	Control (I)*	Control (II)**
				RNA	IENFD
Median age (range)	67.8	64.7	70.9	32.1	60.6
[years]	(54–81)	(54–79)	(58–81)	(22–61)	(51–69)
Number of females/males	7/23	4/11	3/12	14/3	8/7
Median disease duration (range) [years]	11.1	12.3	9.9	N/A	N/A
	(0–22)	(0–22)	(0–22)		
Patients treated with analgesics median age (range) [years]	66	66	N/A	N/A	N/A
	(54–79)	(54–79)			
Number of females/males	2/8	2/8			
Patients not treated with analgesics median age (range)	63.4	63.4	N/A	N/A	N/A
	(54–74)	(54–74)			
Number of females/males	2/3	2/3			
KPPS score (mean± standard deviation)	9.8±8	13.8±8.5	5.4±7.4	N/A	N/A

**Abbreviations:** IENFD = intraepidermal nerve fiber density, KPPS = King’s Parkinson’s Disease Pain Scale, NA/A = not applicable, NRS = Numeric Rating Scale, PD = Parkinson´s disease.

*, **: Control groups used for comparing RNA* and IENFD** data of patients and controls.

### Clinical examination and pain assessment

All patients underwent complete neurological examination and were interviewed about PD-associated pain and prescription of analgesics. The German version of the King’s PD Pain Scale (KPPS) [30], which is the first PD pain-specific scale [31], was used to characterize pain in PD patients and the sum score was calculated as previously published [31, 32]. For pain intensity, the Numeric Rating Scale (NRS) with a range from 0 to 10 (0 = “no pain”, 10 = “worst pain”) was used. Patients were stratified into two subgroups according to previous literature for the NRS [33, 34]: PD Pain and PD No Pain. Per our definition, the PD Pain subgroup included individuals who scored ≥ 4 NRS at the mean pain intensity; the PD No Pain subgroup included those PD patients scoring <4 NRS.

### Skin punch biopsies

Single 5-mm skin punch biopsies (disposable biopsy punch, Kai Medical, Seki, Japan) were obtained from the lateral lower leg of individuals approximately 10 cm above the ankle. Prior to biopsy, the skin was disinfected and then locally anesthetized by subcutaneous injection of 1–2 ml of 10 mg/ml Mecain (PUREN Pharma GmbH & Co., Munich, Germany). Then, skin samples were hemisected: one piece was stored in RNAlater (Invitrogen, Carlsbad, CA, USA) for 24 hours at 4°C and then at -80°C until further processing for gene expression analyses. The second piece was processed for immunohistochemical analysis to determine intraepidermal nerve fiber density (IENFD), as detailed below.

### PGP 9.5 immunofluorescence for IENFD

The hemisected 5-mm skin biopsies were fixed in 4% paraformaldehyde (pH 7.4) for two hours at 4°C, then washed with 0.1 M phosphate-buffered saline (PBS) and stored in 10% sucrose overnight. Thereafter, the samples were embedded in Tissue Tek, frozen in 2-methylbutane cooled in liquid nitrogen and cryo-sectioned into 40μm sections. Sections were then incubated with the pan-neuronal marker protein gene product (PGP) 9.5 antibody (Zytomed, Berlin, Germany) at 1:200 dilution in 0.3% TritonX, 1% Bovine serum albumin in PBS overnight at room temperature.

After washing, Cy3-coupled secondary antibody (109-165-043; 1:50, Dianova, Hamburg Germany) was added and incubated for 2h. After further washing, slides were covered with aqueous media containing DAPI staining, coverslips were added, and they were bordered with nail polish to avoid drying. The IENFD was determined following published guidelines: Briefly, the intraepidermal nerve fibers were manually counted under the microscope (Axiophot 2, Zeiss, Jena, Germany) with a Axiocam camera (Zeiss, Oberkochen, Germany) at 40x magnification.

The epidermal length of each section was measured using Spot 5.2 Advanced Software (SPOT Imaging Solutions, Michigan, IN, USA) [35]. All image analyses were performed with the experimenter blinded to group allocations.

### RNA extraction from skin samples

For processing, RNAlater was removed, skin biopsies were transferred into TRIzol reagent^®^ (Invitrogen, Karlsruhe, Germany), and homogenized using an Ultraturrax homogenizer (Polytron PT 1600E^®^, Kinematica, Luzern, Switzerland). RNA was then isolated using a miRNeasy kit (QIAGEN,Hilden, Germany) following the instructions of the manufacturer. The purity and concentration of RNA was measured using a Nanodrop^®^ spectrophotometer (Peqlab, Erlangen, Germany). Samples were then stored at -80°C.

### cDNA synthesis

For cDNA synthesis, 250 ng of RNA and TaqMan Reverse Transcription Reagents^®^ (Life Technologies, Carlsbad, CA, USA) were used in a final volume of 100 μl. For each reaction, the following were applied: 5 μl of random hexamers, 2 μl oligo-DT, 10 μl 10× buffer, 25 mM MgCl_2_, 20 μl dNTP, 2 μl RNAse inhibitor, 6.2 μl MultiScribe Reverse Transcriptase (50 U/μl). The reaction was then run in an Advanced Primus 96-PCR cycler (Peqlab Biotechnology, Erlangen, Germany). Samples were then stored at -20°C.

### Quantitative real time polymerase chain reaction

The quantitative real time polymerase chain reaction (qRT-PCR) was performed using target-specific TaqMan assays. For each reaction 3.5 μl cDNA, 0.5 μl DNAse free distilled water, 5 μl of TaqMan Fast Advanced Master Mix and 0.5 μl of the specific primer and 0.5 μl of the endogenous control RPL13A (Life Technologies, Carlsbad, CA, USA) were used, giving a total reaction volume of 10 μl per reaction. Samples for each target were run in triplicate, alongside negative controls. The reaction was run using a QuanStudio3 RT-PCR System (Thermo Fisher Scientific, Waltham, MA, USA) under the following conditions: 2 min at 50°C, 2 min at 95°C, 3 sec at 95°C and then 40x cycles of 30 sec at 60°C. The comparative ▵▵Ct method was used to evaluate the acquired data. For each target, a calibrator sample was determined that had a Ct next to the group mean Ct value. The gene-specific Ct value of a sample was normalized to the Ct value of endogenous control, RPL13A and correlated with the above-mentioned calibrator. The correlation is illustrated as 2−▵▵Ct. The Assay-IDs of the primers used are listed in Table 2.

**Table 2:** List of primers used for qRT-PCR analysis.

Target gene	Assay ID
TNF-α	Hs00174128_m1
ΙL-1β	Hs00174097_m1
ΙL-2	Hs00174114_m1
IL-6	Hs00174122_m1
IL-8	Hs00174131_m1
IL-10	Hs00174103_m1
CCL5 (RANTES)	Hs00174086_m1
NF-κB	Hs00765730_m1
SNCA	Hs00240906_m1

**Abbreviations:** CCL5 = C-C chemokine ligand 5, IL = interleukin, NF-κB = nuclear factor kappa B, RANTES = regulated on activation, normal T cell expressed and secreted, qRT-PCR = quantitative real-time polymerase chain reaction, SNCA = synuclein alpha, TNF-α = tumor necrosis factor alpha.

### Data handling and statistical analysis

Samples with ambiguous and below the level of detection Ct values were excluded from the analysis. All data were expressed as median and range and analysed using nonparametric Mann-Whitney test or Kruskal–Wallis followed by Dunn’s multiple comparisons, as appropriate. Supplementary data were analyzed using the Pearson’s test or Two-way ANOVA followed by Sidak’s multiple comparisons, as appropriate. Significance was set at the 5% confidence level for all analyses and performed using SPSS software 27 version (IBM, Chicago, IL, USA). Graphs were drawn with GraphPad Prism 9.00 (GraphPad, San Diego, CA, USA) for Windows 10.

## Results

### IENFD in the skin is lower in PD compared to healthy controls

**Fig 1:**
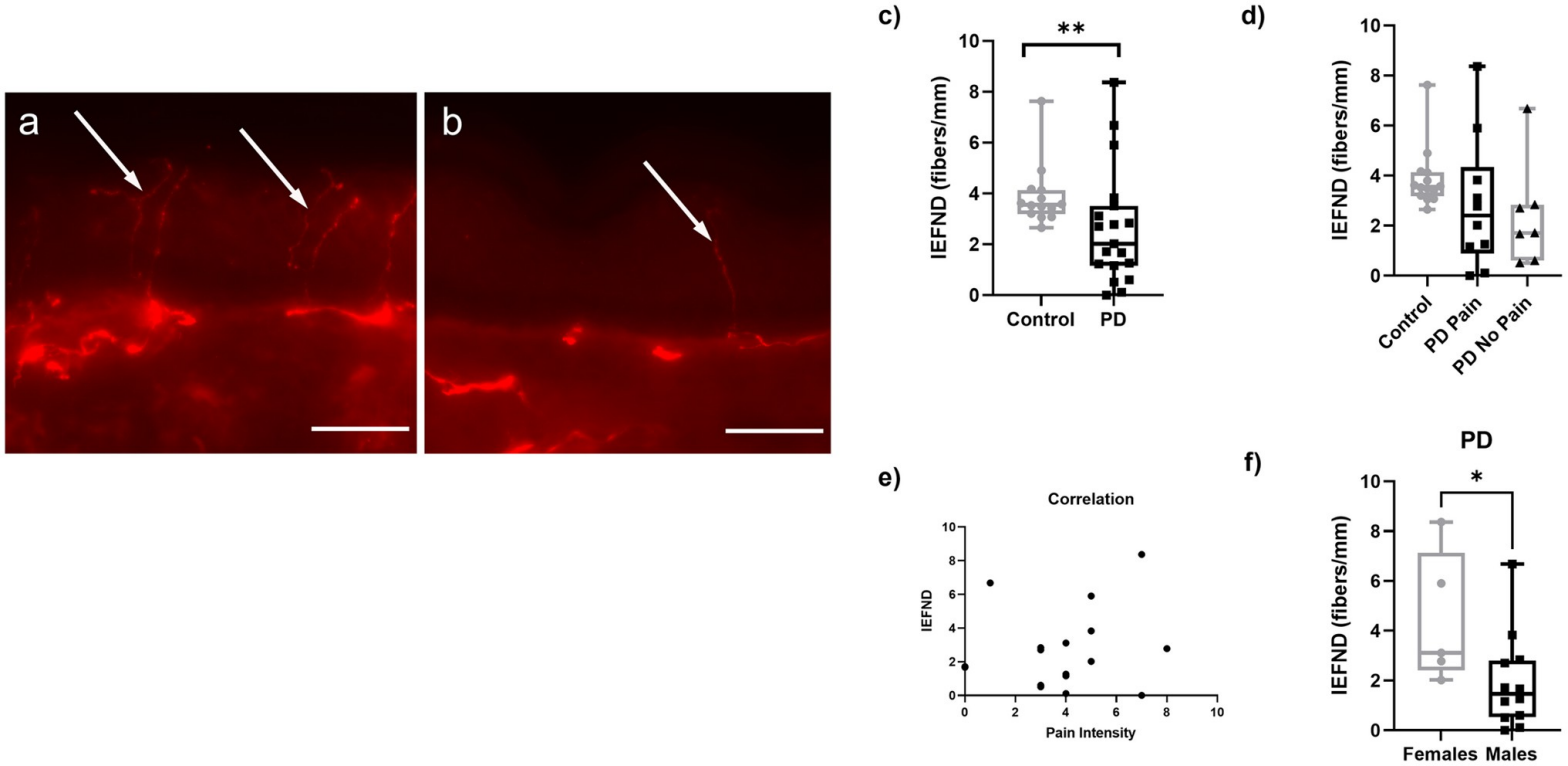
Quantification of intraepidermal nerve fiber density in skin punch biopsies obtained from the lateral lower leg. Representative images of immunohistochemical staining using PGP9.5 (red) antibody as a pan-neuronal marker in 40-μm sections from the skin biopsy of a healthy control showing multiple intraepidermal nerve fibers (arrows). A representative biopsy from a healthy control (a) and a patient with PD (b); scale bars = 50 μm. Quantification shows lower IENFD in PD compared to healthy control skin biopsies (c). No intergroup differences for the IENFD between PD Pain and PD No Pain patients (d). No correlation of IENFD and patients`pain intensity (e). Lower IENFD in women with PD compared to men with PD (f). Data are expressed as median and range. Abbreviations: IENFD = intraepidermal nerve fiber density, PD = Parkinson’s disease, PGP9.5 = protein gene product 9.5. *P<0.05, **P<0.01. Mann-Whitney-U test.

Firstly, we assessed whether IENFD is different between PD and healthy controls. We observed lower counts in the PD group (n = 19) compared to healthy controls (n = 14) (p<0.01) (Fig 1a–1c). However, when comparing the two PD subgroups, no difference was found between PD Pain patients and PD No Pain patients (Fig 1d). Consistent with this, no correlation was observed for IENFD and pain intensity in the PD group (Fig 1e). Men with PD had lower IENFD compared to women with PD (p<0.05) (Fig 1f).

### In skin biopsies of PD patients, IL-2 and TNF-α gene expression is higher compared to healthy controls

**Fig 2:**
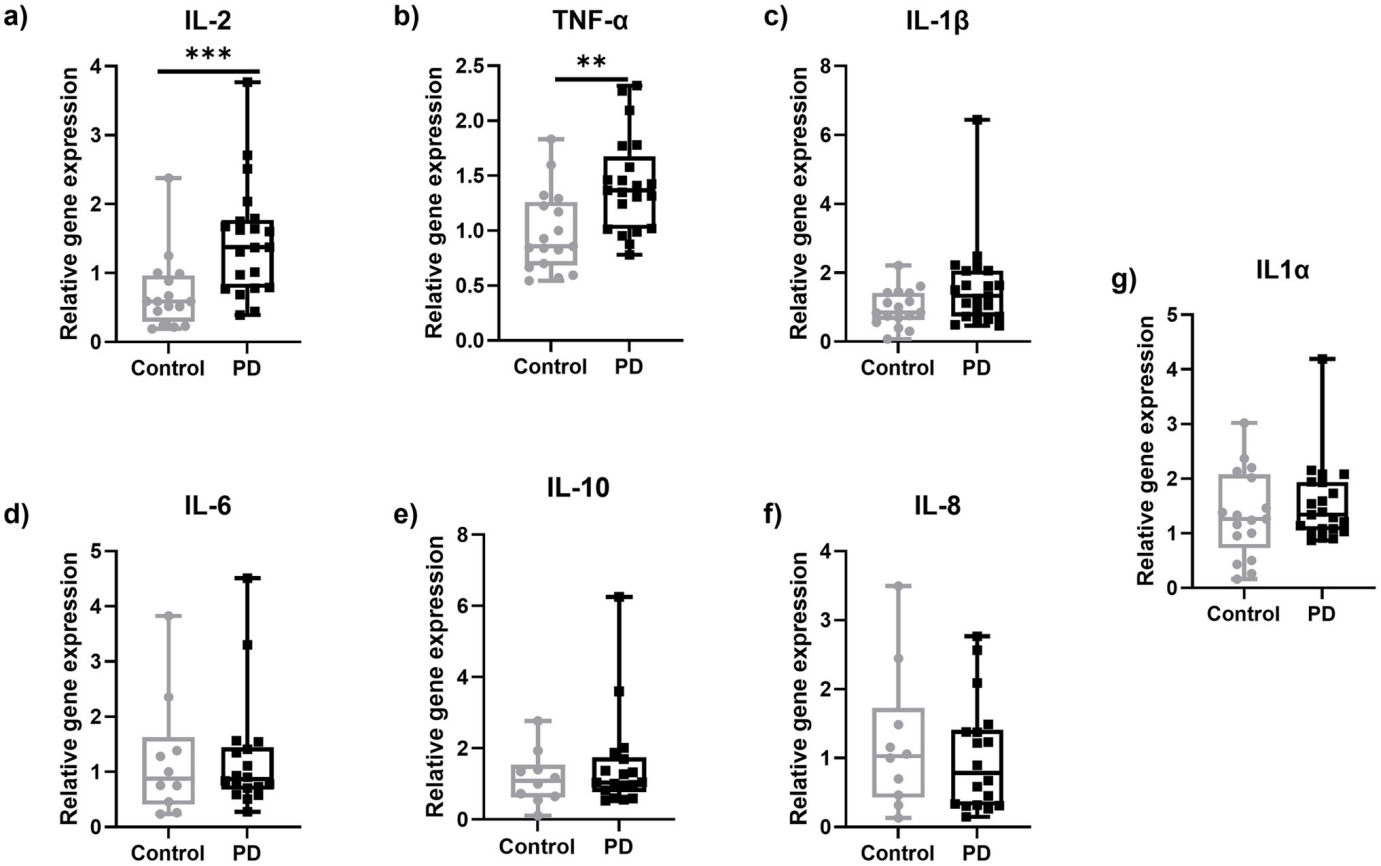
Gene expression of selected proinflammatory cytokines in skin punch biopsy samples obtained from the lateral lower leg of PD patients and healthy controls. Higher gene expression of IL-2 (a) and TNF-α (b) in PD skin biopsies compared to healthy controls. No intergroup differences were found for the investigated markers IL-1β (c), IL-6 (d), IL-10 (e), IL-8 (f), and IL-1α (g). Data are expressed as median and range. Abbreviations: IL = interleukin, PD = Parkinson’s disease, TNF-α = tumor necrosis factor-alpha. **P<0.01, ***P<0.001. Mann-Whitney-U test.

We found higher expression of the proinflammatory cytokines IL-2 (p<0.001) and TNF-α (p<0.01) in skin samples of patients with PD compared to healthy controls (Fig 2a and 2b). The expression of these two cytokines did not correlate with age in both groups (S1 and S2 Figs) and no differences were observed between men and women in both groups (S4 Fig).

**Fig 3:**
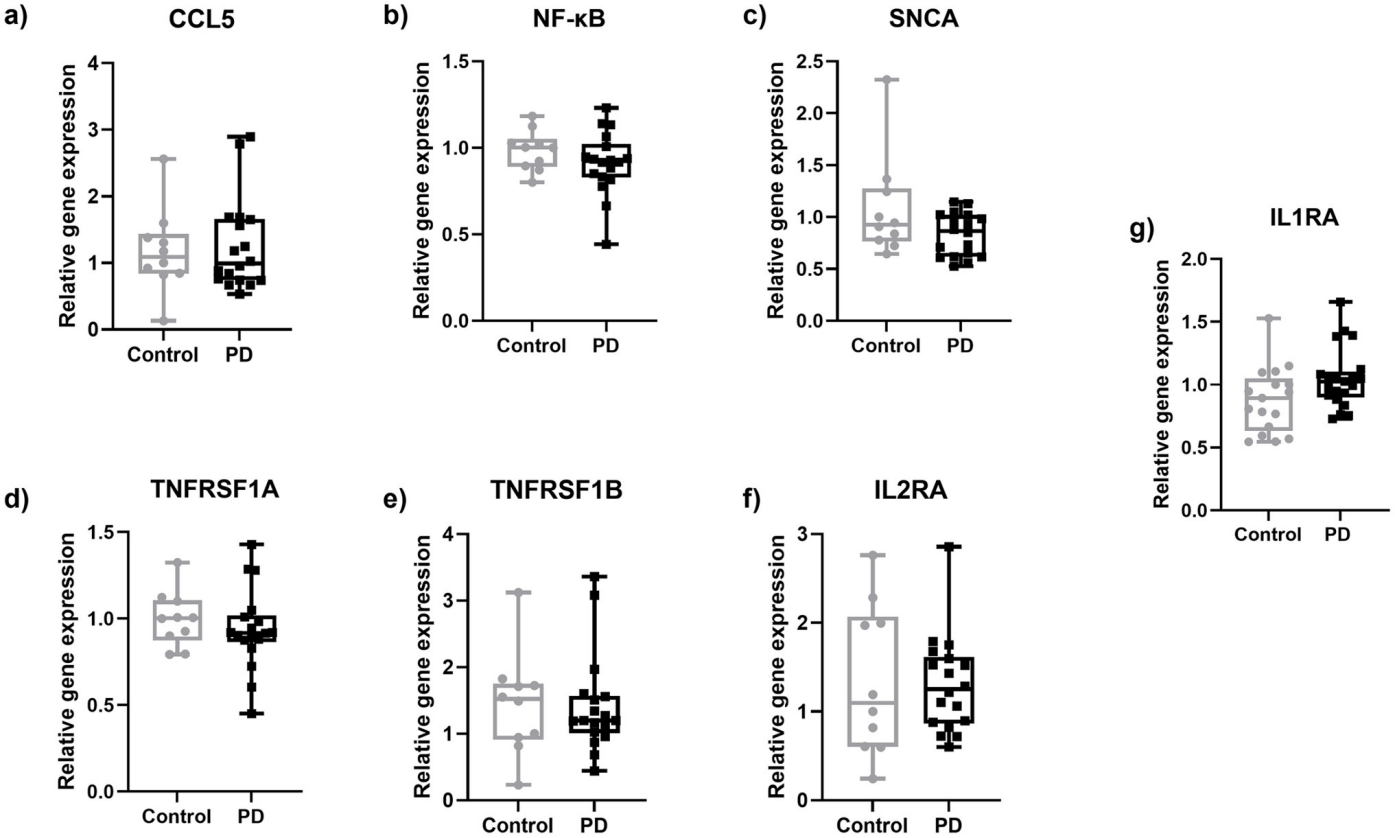
Gene expression of selected markers in skin punch biopsy samples obtained from the lateral lower leg of PD patients and healthy controls. No intergroup differences were found for the investigated markers CCL5 (a), NFκB (b), SNCA (c), TNFRSF1A (d), TNFRSF1B (e), IL2RA (f), and IL1RA (g) in PD skin biopsies compared to healthy controls. Data are expressed as median and range. Abbreviations: CCL5 = C-C chemokine ligand 5, IL1RA = interleukin 1 receptor A, IL2RA = interleukin 2 receptor A, NFκB = nuclear factor kappa B, PD = Parkinson’s disease, SNCA = synuclein alpha, TNFRSF1A = TNF receptor superfamily member 1A, TNFRSF1B = TNF receptor superfamily member 1B. Mann-Whitney-U test.

All other cytokines investigated did not differ between groups (Fig 2c–2g). The investigation of TNF receptor superfamily member 1A/B (TNFRSF1A/B), IL2 receptor (IL2R), nuclear factor kappa B (NFKΒ), and synuclein alpha (SNCA) in skin samples of PD patients and healthy controls did not show intergroup differences (Fig 3).

### In skin biopsies of PD patients with pain, IL-1β gene expression is higher compared to healthy controls

**Fig 4:**
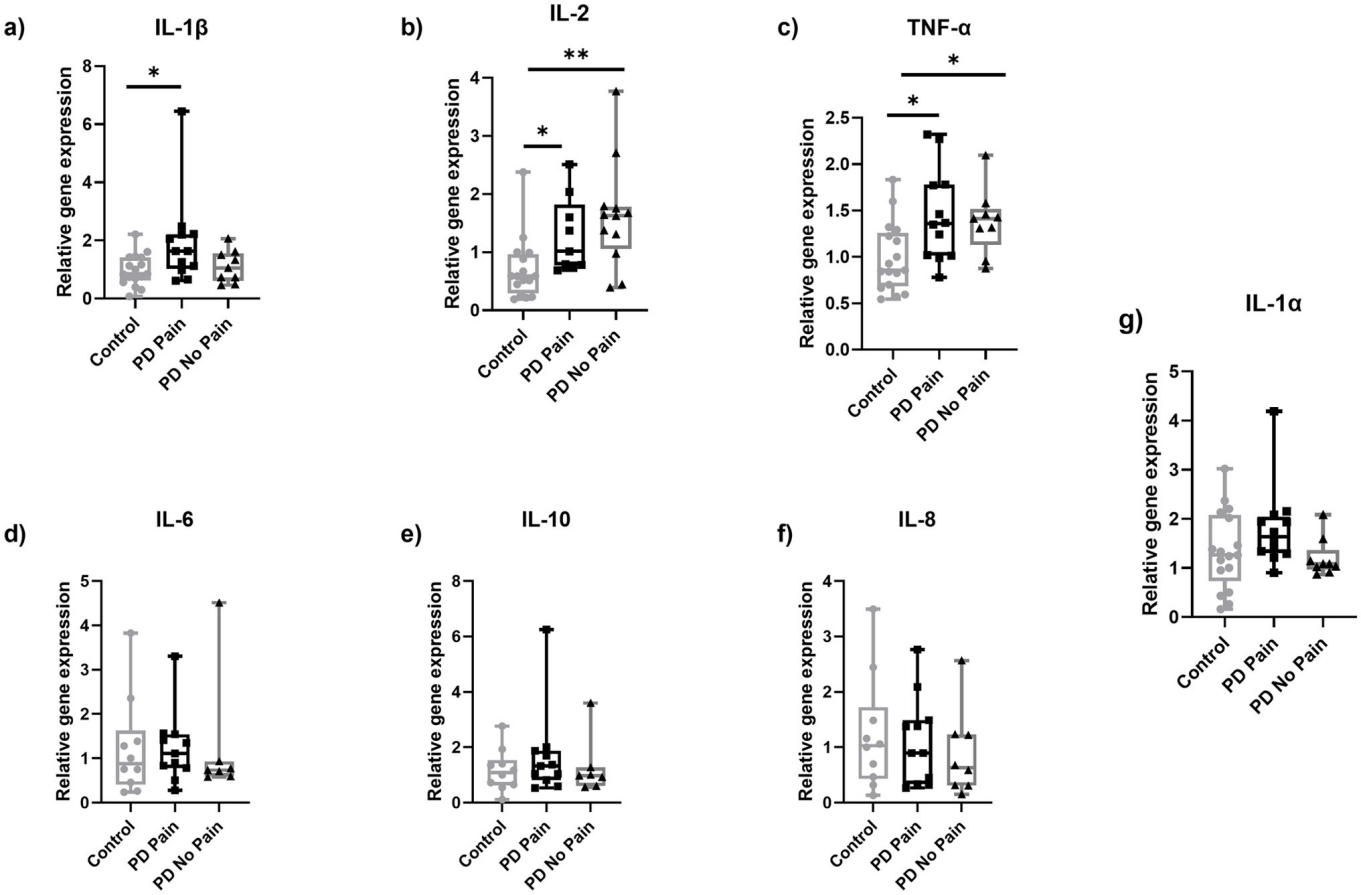
Gene expression of proinflammatory mediators in skin punch biopsy samples obtained from the lateral lower leg of PD Pain patients, PD No Pain patients, and healthy controls. IL-1β gene expression was higher in skin samples of PD Pain patients compared to controls (a). IL-2 (b) and TNF-α (c) expression was higher in skin samples of PD Pain and PD No Pain patients compared to controls without intergroup difference. No intergroup differences were found for the investigated markers IL-6 (d), IL-10 (e), IL-8 (f), and IL-1α (g). Data are expressed as median ± range. Abbreviations: IL = interleukin, PD = Parkinson’s disease, TNF-α = tumor necrosis factor alpha. *P<0.05. Kruskal Wallis followed by Dunn’s multiple comparisons.

We next compared the gene expression of inflammatory mediators between the PD subgroups and the healthy controls. Consistent with the above findings for the PD group as a whole, IL-2 and TNF-α gene expression was higher in both PD pain and PD No Pain subgroups compared to healthy controls. However, there was no difference between the PD Pain and PD No Pain subgroups (Fig 4a and 4b). In addition, we observed a higher expression of IL-1β in the skin of patients in the PD Pain group compared to healthy controls (p<0.05) (Fig 4c). IL-1β gene expression did not correlate with an increase in age the PD and healthy control groups (S3 Fig). Moreover, the expression did not differ between men and women (S4 Fig).

**Fig 5:**
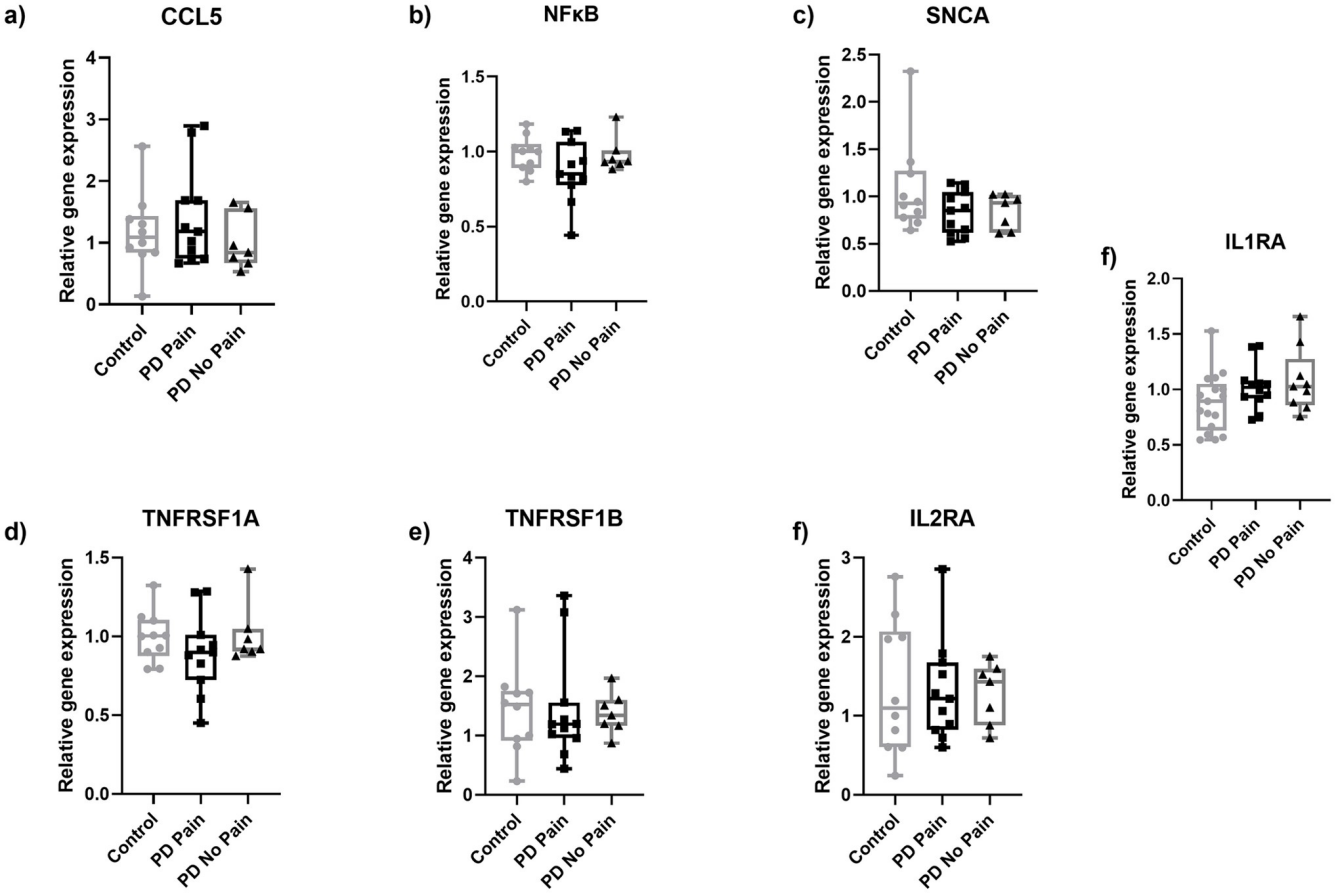
Gene expression of inflammatory mediators in PD Pain skin punch biopsies obtained from the lateral lower leg. No changes in in the expression of CCL5 (a), NFκB (b), SNCA (c), TNFRSF1A (d), TNFRSF1B (e), IL2RA (f), and IL1RA (g) in PD with pain skin biopsies compared to healthy controls. Data are expressed as median ± range. Abbreviations: CCL5 = C-C chemokine ligand 5, IL2RA = interleukin 2 receptor A, NFκB = nuclear factor kappa B, PD = Parkinson’s disease, SNCA = synuclein alpha, TNFRSF1A = TNF receptor superfamily member 1A, TNFRSF1B = TNF receptor superfamily member 1B. Kruskal Wallis followed by Dunn’s multiple comparisons.

We did not detect a difference when comparing the PD No Pain group to either the healthy controls, or to the PD Pain group. All other cytokines and inflammatory markers investigated did not differ between groups (Figs 4d–4g and 5).

### In the skin of PD patients with pain, TNF-α gene expression is lower in those treated with analgesics compared to those without

**Fig 6:**
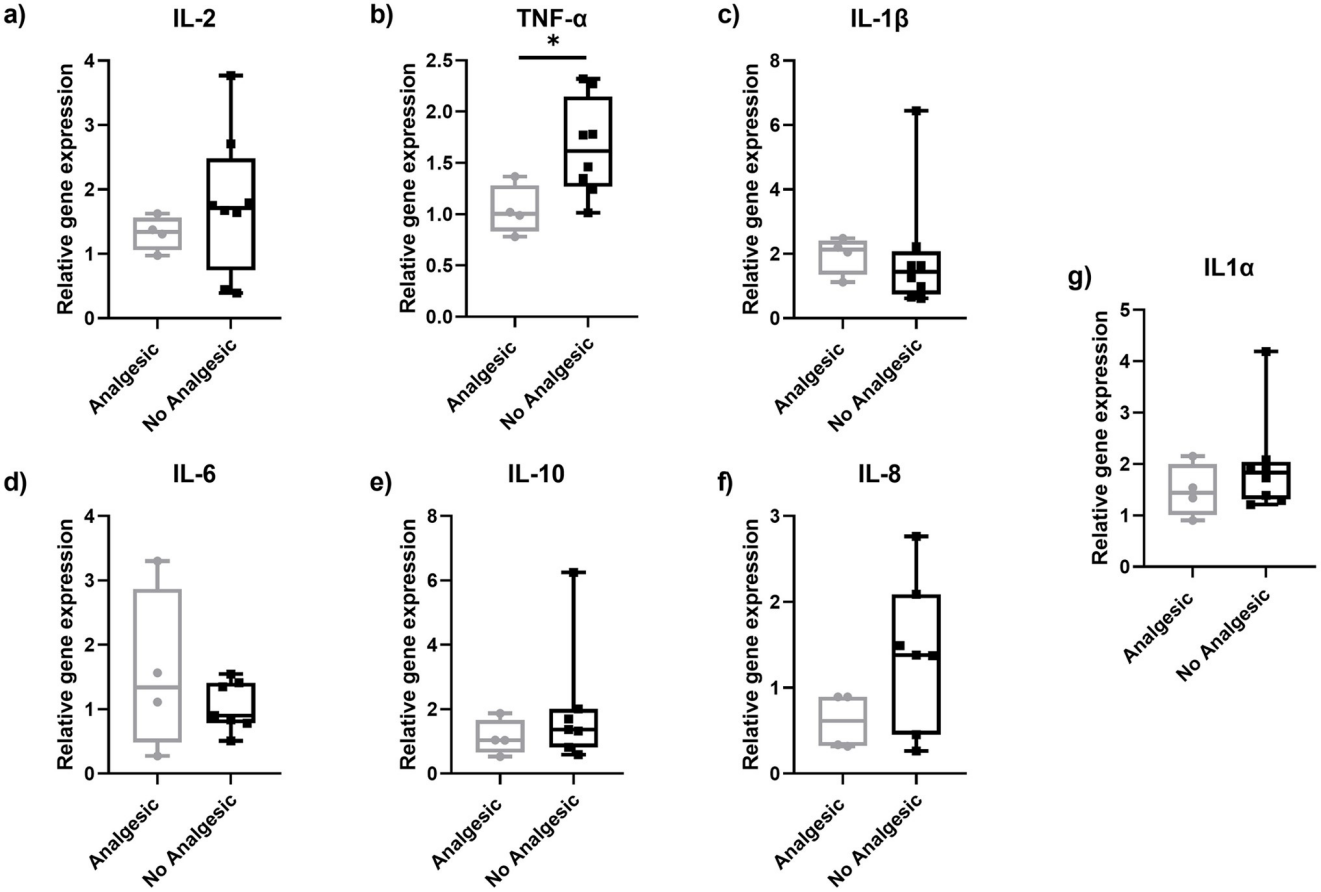
Gene expression of inflammatory mediators in PD Pain treated with analgesics skin punch biopsies obtained from the lateral lower leg. Lower gene expression of TNF-α in PD with pain treated with analgesics compare to those not treated (b). No intergroup differences were found for the investigated markers IL-2 (a), IL-1β (c), IL-6 (d), IL-10 (e), IL-8 (f), and IL-1α (g). Data are expressed as median ± range. Abbreviations: IL = interleukin, PD = Parkinson’s disease, TNF-α = tumour necrosis factor alpha. *P<0.05. Mann-Whitney-U test.

**Fig 7:**
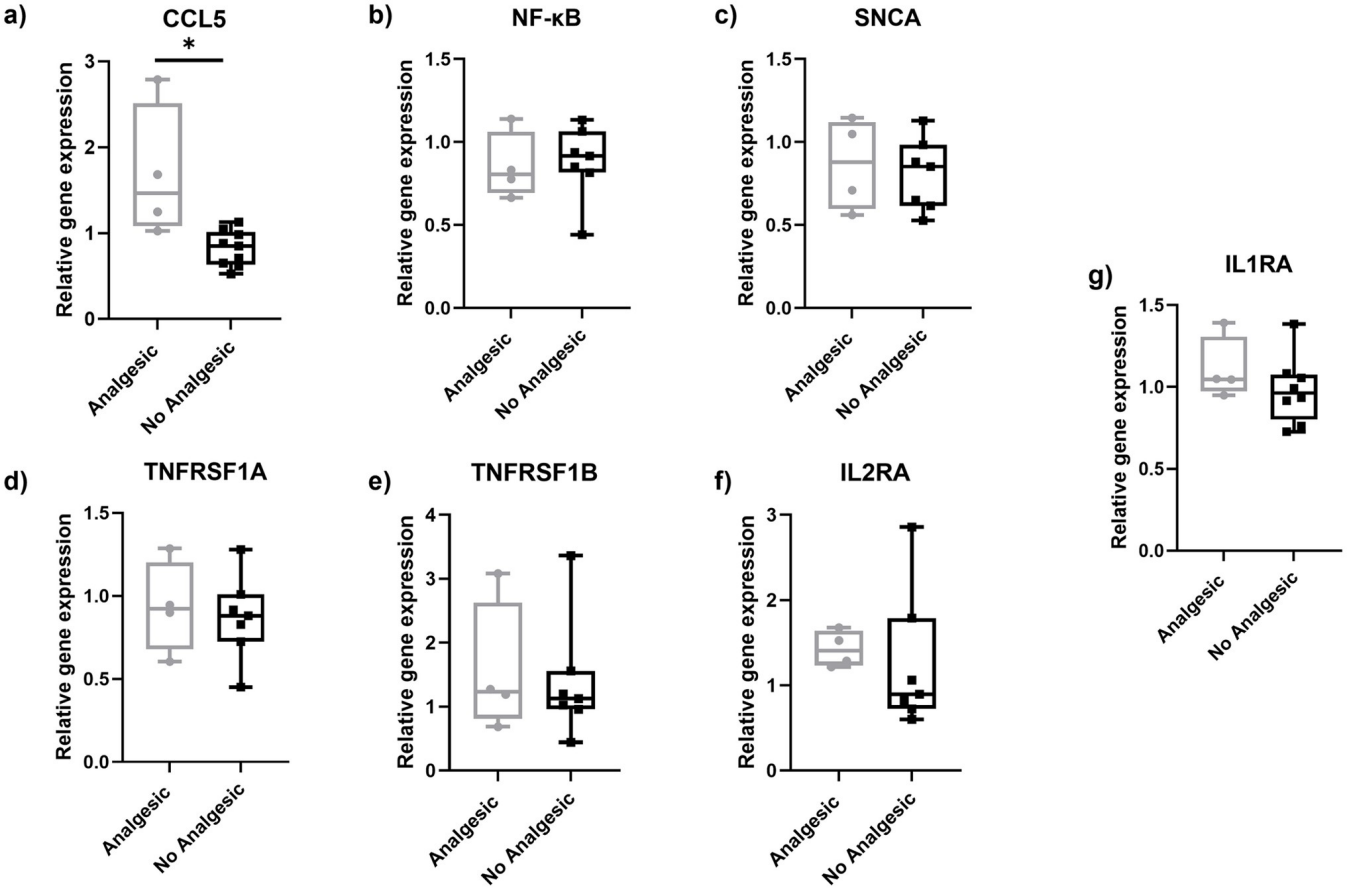
Effect of analgesic treatment in gene expression of inflammatory markers in PD Pain skin punch biopsies obtained from the lateral lower leg. Higher gene expression of CCL5 in PD with pain treated with analgesics compare to those not treated. No intergroup differences were found for the NFκB (b), SNCA (c), TNFRSF1A (d), TNFRSF1B (e), IL2RA (f), and IL1RA (g). Data are expressed as median ± range. Abbreviations: CCL5 = C-C chemokine ligand 5, IL2RA = interleukin 2 receptor A, NFκB = nuclear factor kappa B, PD = Parkinson’s disease, SNCA = synuclein alpha, TNFRSF1A = TNF receptor superfamily member 1A, TNFRSF1B = TNF receptor superfamily member 1B. *P<0.05. Mann-Whitney-U test.

In order to understand whether the expression of inflammatory markers is different in patients with PD Pain prescribed with analgesics compared to those not, we further divided the PD Pain group into Analgesic and No Analgesic subgroups. Four of the patients experiencing pain received one or a combination of the following drugs as analgesics: ibuprofen, oxycodone/naloxone, pregabalin, paracetamol, metamizol, and diclofenac daily. We found a lower gene expression of TNF-α (p<0.05) (Fig 6b) and a higher expression of C-C chemokine ligand 5 (CCL5) (p<0.05) (Fig 7a) in the Analgesic group compared to the No Analgesic group. The investigation of all other markers did not show intergroup differences (Figs 6 and 7).

## Discussion

In this study, we investigated whether changes in skin innervation and cutaneous expression of inflammatory markers are present in PD patients and whether such changes could explain the pain experienced by some. We found evidence for reduced IENFD and higher gene expression of IL-2 and TNF-α in the skin of patients with PD, irrespective of their pain status, while IL-1β expression was higher only in the skin of PD patients suffering from pain than in healthy controls.

The lower IENFD in skin punch biopsies of PD patients compared to healthy controls is in accordance with previous reports [17, 18, 21, 22, 36], supporting the validity of our data. Consistent with findings in the general population [37, 38], we also found lower IEFND in male PD patients versus females. We extended these previous findings to investigate whether the reduction in IENFD had a potential link to pain in PD. However, we found no correlation between IENFD and pain in PD, indicating that the denervation underpinning reduced IEFND may not be associated with the development and maintenance of pain in PD. Though still a matter of debate [33, 39–42], our findings are in line with what was reported in studies of non-PD related peripheral neuropathies, where the loss of intraepidermal nerve fibers did not correlate with pain. However, it is important to note that by monitoring IENFD via PGP9.5 immunofluorescence, which picks all fiber types, we cannot exclude potential correlations of pain with changes in distinct fiber subtypes. Future quantitative studies investigating the different subtypes of nerve fibers in combination with tests assessing fiber function and conduction such as quantitative sensory testing and laser evoked potentials will contribute to the delineation of correlations between PD pain and the dysfunction or loss of specific fibers.

Elevated expression of proinflammatory cytokines in the skin may lead to peripheral sensitization and pain [25]. Higher expression of IL-1β in skin samples of the PD Pain group compared to healthy controls may indicate an involvement of IL-1β in PD related pain. Li et al. similarly observed elevated systemic IL-1 in a PD cohort experiencing pain compared to a healthy control group pointing towards an involvement of IL-1 in PD pain [28]. In further support of a role for IL-1β in pain itself, increased production of IL-1β was shown to lead to hypersensitivity in an animal model of chronic pain [43]. Moreover, IL-1β stimulates the expression of cyclooxygenase (COX)-2 [44] and inhibition of COX-2 underlies the analgesic effects of non-steroidal anti-inflammatory drugs (NSAIDs) [45]. Amongst many analgesics, NSAIDs were prescribed in our cohort, however, the expression of IL-1β was not different between those people prescribed analgesics and those not.

The reason underlying the elevated expression of proinflammatory cytokines in the skin of PD patients is not well understood. α-synuclein is known to trigger the expression of proinflammatory cytokines such as IL-1β *in vitro* [46] and α-synuclein deposits have been found in the skin of PD patients [23], supporting these as a possible trigger for the elevated IL-1β levels in skin of PD patients with pain, noted in our study. Although α-synuclein (*SNCA*) gene expression was not increased in the skin of PD patients with pain, whether protein levels themselves are increased or whether post transcriptional modification of α-synuclein occurs (e.g., phosphorylation) remains to be investigated. As a result, α-synuclein cannot be excluded as a potential driver of the elevated expression of the inflammatory mediator IL-1β in the skin of PD patients with pain.

Though not correlated with pain seen in PD, IL-2 and TNF-α were elevated in the skin of PD patients compared to healthy controls, indicating a potential involvement in PD peripheral pathophysiology. The higher levels of IL-2 in the skin of PD compared to healthy controls, were not accompanied by increased expression of IL2R which is the only known receptor of IL-2, but if translated into increased IL-2 protein, would provide increased substrate to potentially increase activation [47]. IL-2 is produced mainly by dendritic cells and T-cells and is associated with an activated state of T-cells [48, 49]. Alterations in T-cells populations have been previously described in PD patients [50, 51], as well as the presence of α-synuclein specific T-cells [52]. A possible explanation of the increased IL-2 in the skin of PD patients could be the presence of activated T-cells, as α-synuclein inclusions are found in the skin of PD patients [23, 36, 53]. TNF-α is one of the cytokines that were increased in the PD group alongside IL-2. Activated CD4^+^ T-cells produce and secrete IL-2 and can then be differentiated in several subtypes such as Th1 and Th17 which are pro-inflammatory and Th2 and regulatory T-cells which are anti-inflammatory [54]. Each subgroup produces a different mix of cytokines. Of particular note, Th1 cells are producing TNF-α, IL-2, and IFN-γ. Given that IL-2 and TNF-α were elevated in PD biosamples, a potential source of these cytokines in our samples could be Th1 cells. This suggestion is in accordance with studies in the periphery showing a higher percentage of Th1 cells in PD patients [55]. Future studies examining the type and presence of T-cells in the skin of PD patients are required to test this hypothesis. TNF-α acts via its two receptors, TNF receptor 1 (TNFR1), and TNFR2 which are encoded by the TNFRSF1A and TNFRSF1B genes, respectively. When TNF-α binds to its receptors, it activates the production of NF-κB [56]. Despite seeing elevated TNF-α gene expression, we did not observe a difference in the transcriptional expression of either TNFRSF1A or TNFRSF1B. In addition, we did not observe any increase in NF-κΒ, indicating that this specific signalling pathway is not activated in the skin in PD. However, an involvement of TNF-α in the manifestation of pain in PD cannot be completely excluded, as patients treated with analgesics in our PD Pain cohort had significantly lower expression of TNF-α in the skin compared to those not receiving treatment.

There are some potential limitations to our study that require consideration. Firstly, changes in gene expression do not always correspond to the same changes in protein levels, while some cytokines are also produced as precursors and cleavage, or post-transcriptional modification is required for activation. However, measuring the protein expression of cytokines in the skin is challenging as cytokines are unstable with a short half-life and a high susceptibility to post-sampling handling. Nevertheless, gene expression does provide valuable insight into the potential changes in cytokine expression. Secondly, the PD and control groups also differed in age, with the PD group being older than the healthy control group. Aging has been associated with alterations in the immune system with immunosenescence being evident in the elderly [57]. While cytokine expression has been extensively studied in plasma and serum of healthy individuals over a wide age range, the number of studies measuring the cutaneous expression of cytokines is limited [58–61]. The age of our healthy control group was lower compared to our PD group. However, we found no correlation of age with cytokine expression in the skin in both groups suggesting that the alterations we observed are unlikely a result of diversity in age. Similar findings have been reported by other groups, where comparisons of TNF-α, IL-2, and IL-1β expression in the skin between different age groups did not reveal any differences [62–64]. Another study measuring TNF-α mRNA expression in the skin of healthy individuals from different age groups revealed no differences between young (18–35 years old) versus old (76–88 years old) [65]. In a further study, the authors showed that levels of T-cell-derived IL-2 in the skin did not differ between young (<40 years old) and old (>60 years old) subjects [63]. Finally, examinations of TNF-α and IL-2 in three different age groups (21–27, 52–64, 70–77 years old) also revealed similar levels of expression in each [64]. Hence, we are confident that the age gap between our study cohorts has minor impact on the presented data, if any. Thirdly, the sex composition was different in the control (more women) and PD group (more men), however, this does not seem to have contributed to the changes we observed, as the levels of cytokines were similar between men and women in our control and PD cohorts. Studies comparing inflammatory responses between men and women have shown that expression of inflammatory cytokines is increased in women with age [66]. Given that the cytokines (TNF-α and IL-2) were higher in the PD group, which consisted mainly of men, sex is unlikely to have been a determining factor of this result. Fourthly, another limitation of our study is the use of two different groups of healthy controls for IEFND count and RNA analysis, which may have added variability to our data. However, the inclusion criteria were the same for both control groups. Healthy controls underwent clinical examination and only individuals without an ongoing inflammation and symptoms of a neuropathy were included. Finally, given our PD cohort was on antiparkinsonian medication during the study, we cannot exclude the possibility that our results were impacted by treatment as well as PD itself. More specifically, dopaminergic treatment is known to alter the immune response of PD patients, as dopaminergic receptors are expressed on immune cells in the periphery [67–69]. Treatment, with L-DOPA has been partially associated with increased levels of cytokines, such as CCL5 in the serum of PD patients [70]. Interestingly, a study of 21 no previously treated PD patients showed increased levels of IL-2 in the serum which was reversed post-L-DOPA administration [71]. However, whether similar alterations are reflected in the skin, remains to be elucidated in future studies by including untreated patients.

## Conclusion

In conclusion, our data demonstrate a higher expression of cutaneous IL-2 and TNFα in patients with PD compared to healthy controls independent of skin innervation and pain phenotype. Our data indicate the occurrence of a local, peripheral inflammatory response in the skin in PD, but do not support this being a contributing factor to the expression of pain in PD.

## Supporting information

S1 Fig TNF-α expression and age in skin punch biopsies of healthy controls and PD patients. Age is not correlated with altered TNF-α gene expression in the skin punch biopsy samples obtained from the lateral lower leg of healthy controls (a, b) and PD patients (c, d). Abbreviations: PD = Parkinson’s disease, TNF-α = tumor necrosis factor-alpha. Pearson’s correlation analysis.
(TIF)

S2 Fig IL-2 expression and age in skin punch biopsies of healthy controls and PD patients. Age is not correlated with altered ΙL-2 gene expression in the skin punch biopsy samples obtained from the lateral lower leg of healthy controls (a, b) and PD patients (c, d). Abbreviations: IL = interleukin, PD = Parkinson’s disease. p > 0.05 Pearson’s correlation analysis.
(TIF)

S3 Fig IL-1β expression and age in skin punch biopsies of healthy controls and PD patients. Age is not correlated with altered IL-1β gene expression in the skin punch biopsy samples obtained from the lateral lower leg of healthy controls (a, b) and PD patients (c, d). Abbreviations: IL = interleukin, PD = Parkinson’s disease. p > 0.05 Pearson’s correlation analysis.
(TIF)

S4 Fig No sex differences in the gene expression of selected cytokines in the skin punch biopsy samples obtained from the lateral lower leg of healthy controls and PD patients. TNF-α (a, b), IL-2 (c, d), and IL-1β (e, f) gene expression did not differ between men and women in the healthy control and PD groups. Abbreviations: IL *=* interleukin, PD *=* Parkinson*’*s disease. Pearson*’*s correlation analysis, TNF-α *=* tumour necrosis factor alpha. P > 0.05. Two-Way ANOVA followed by Sidak*’*s multiple comparisons.
(TIF)
