# Supplementary figures and images for: Proinflammatory profile in the skin of Parkinson’s disease patients with and without pain
Source: PLoS One. 2022 Oct 27;17(10):e0276564. doi: 10.1371/journal.pone.0276564 (PMC9612575; doi:10.1371/journal.pone.0276564)

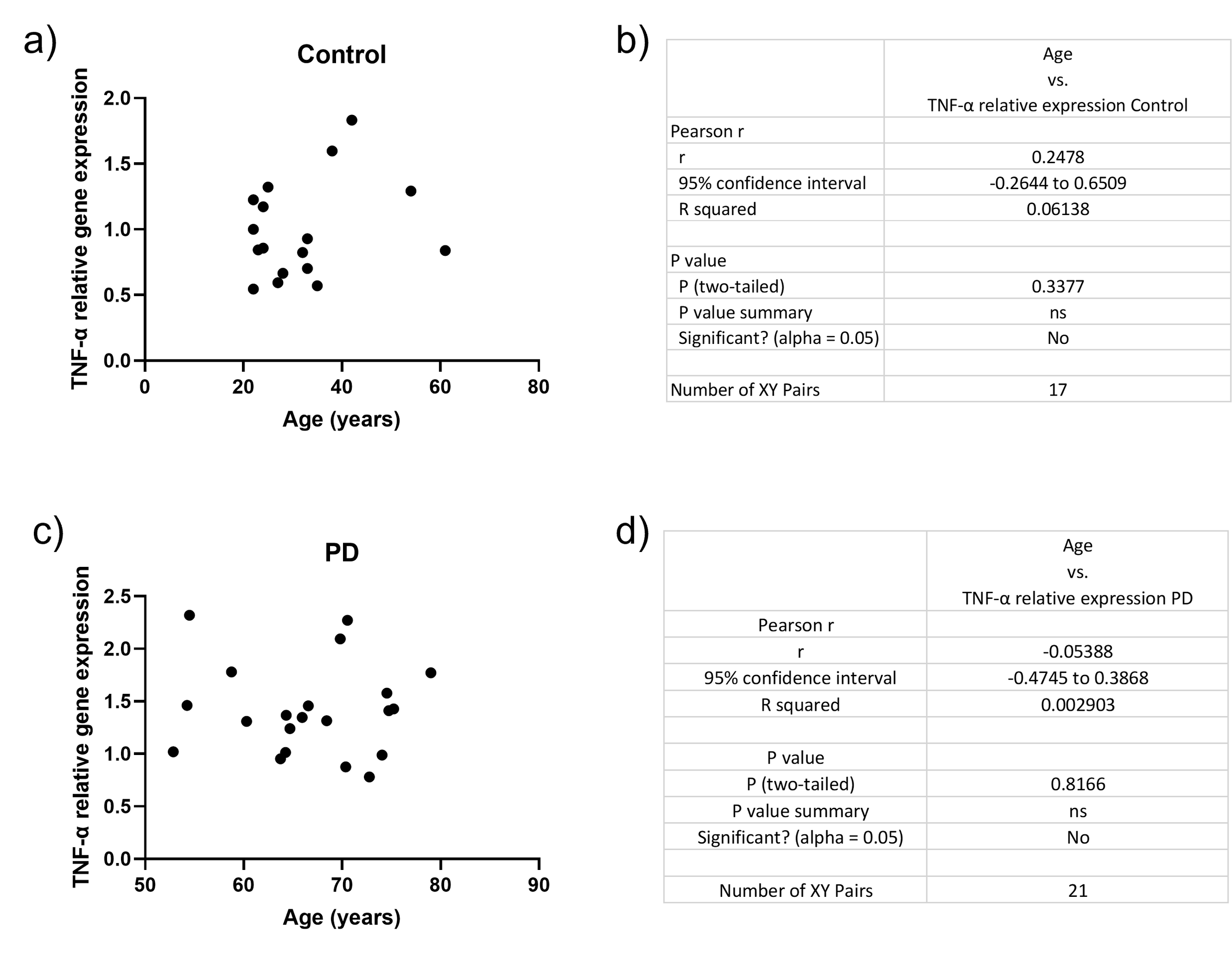

Supplement: S1 Fig — Age is not correlated with altered TNF-α gene expression in the skin punch biopsy samples obtained from the lateral lower leg of healthy controls (a, b) and PD patients (c, d). Abbreviations: PD = Parkinson’s disease, TNF-α = tumor necrosis factor-alpha. Pearson’s correlation analysis. (TIF) [file pone.0276564.s001.tif]

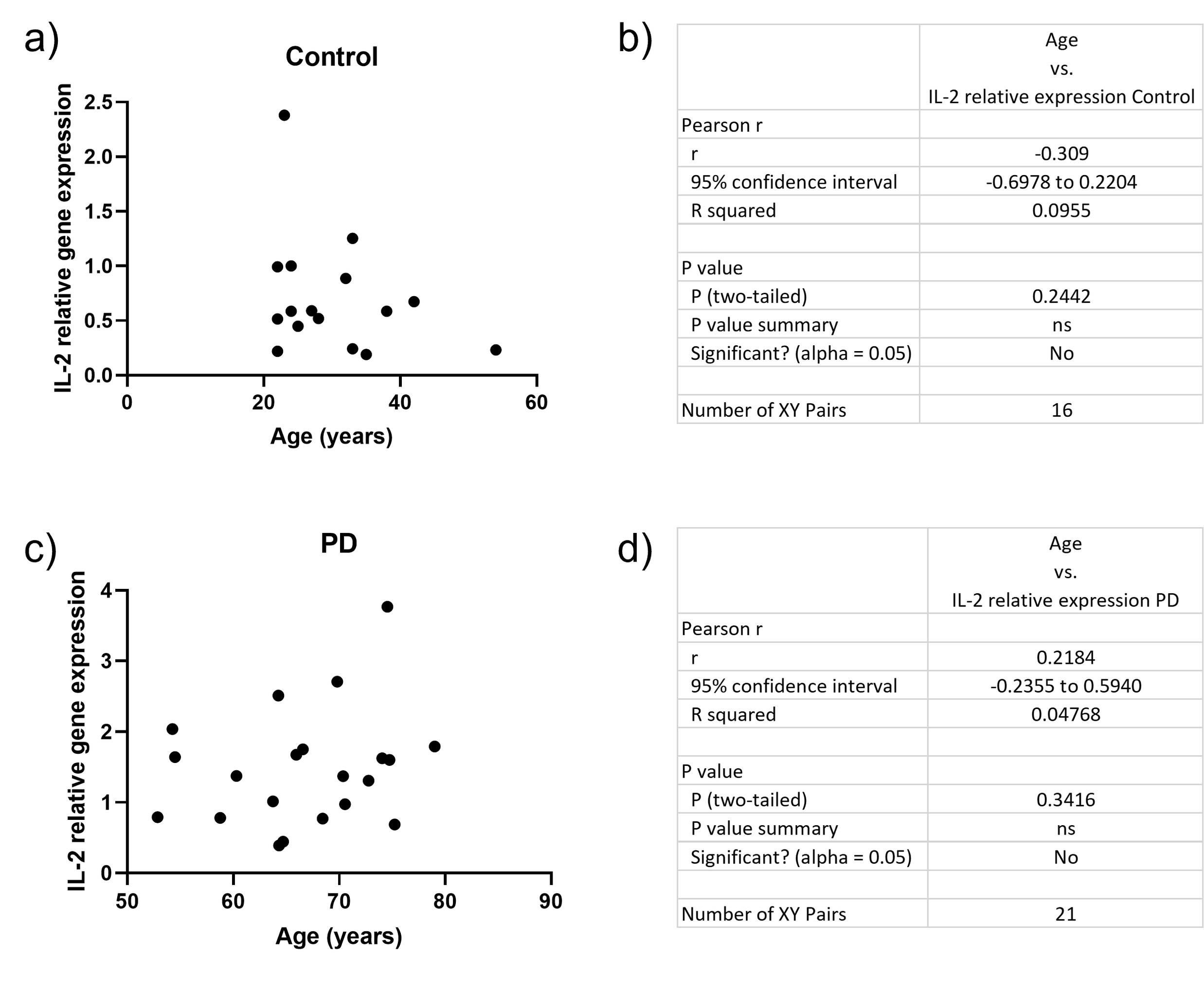

Supplement: S2 Fig — Age is not correlated with altered ΙL-2 gene expression in the skin punch biopsy samples obtained from the lateral lower leg of healthy controls (a, b) and PD patients (c, d). Abbreviations: IL = interleukin, PD = Parkinson’s disease. p > 0.05 Pearson’s correlation analysis. (TIF) [file pone.0276564.s002.tif]

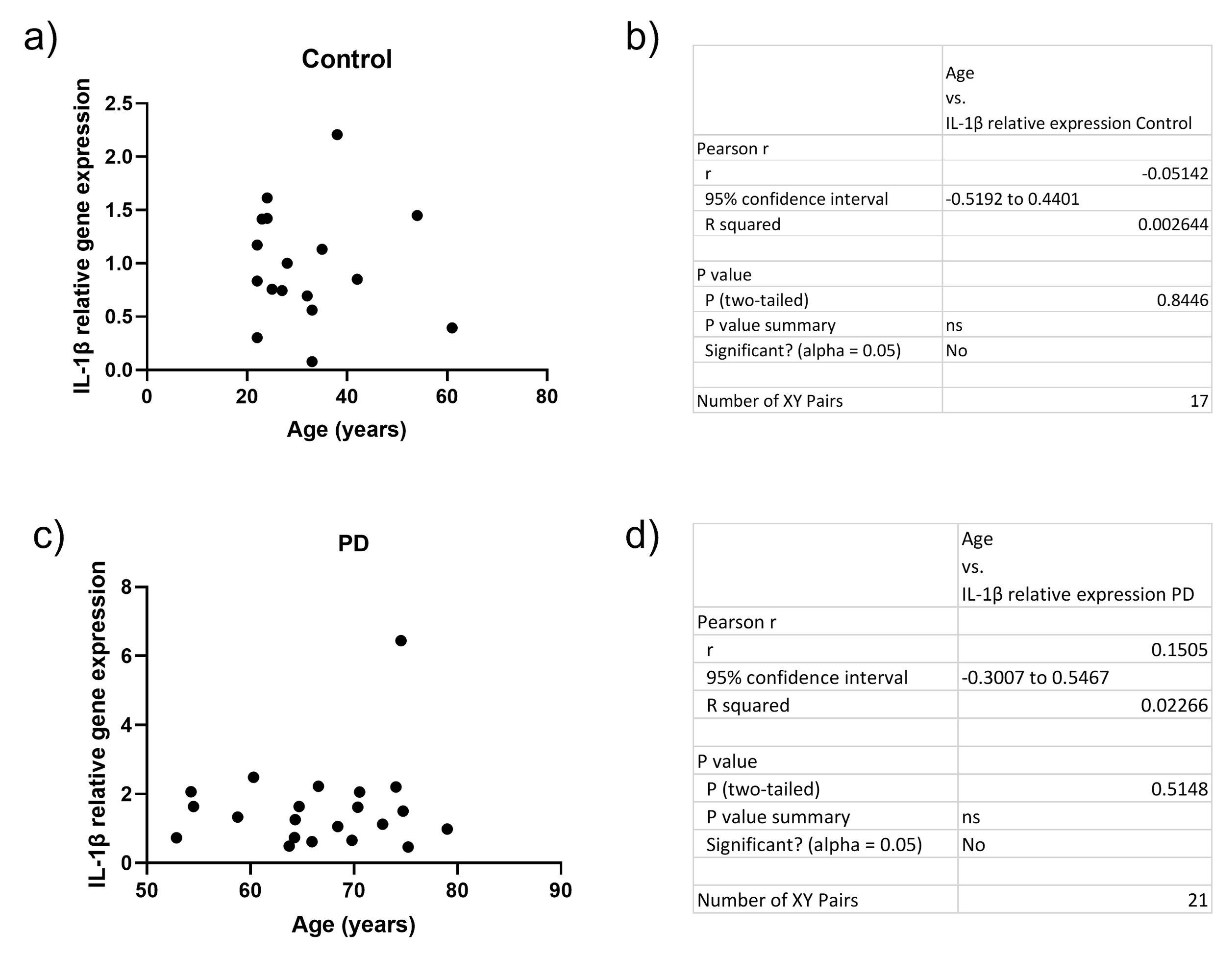

Supplement: S3 Fig — Age is not correlated with altered IL-1β gene expression in the skin punch biopsy samples obtained from the lateral lower leg of healthy controls (a, b) and PD patients (c, d). Abbreviations: IL = interleukin, PD = Parkinson’s disease. p > 0.05 Pearson’s correlation analysis. (TIF) [file pone.0276564.s003.tif]

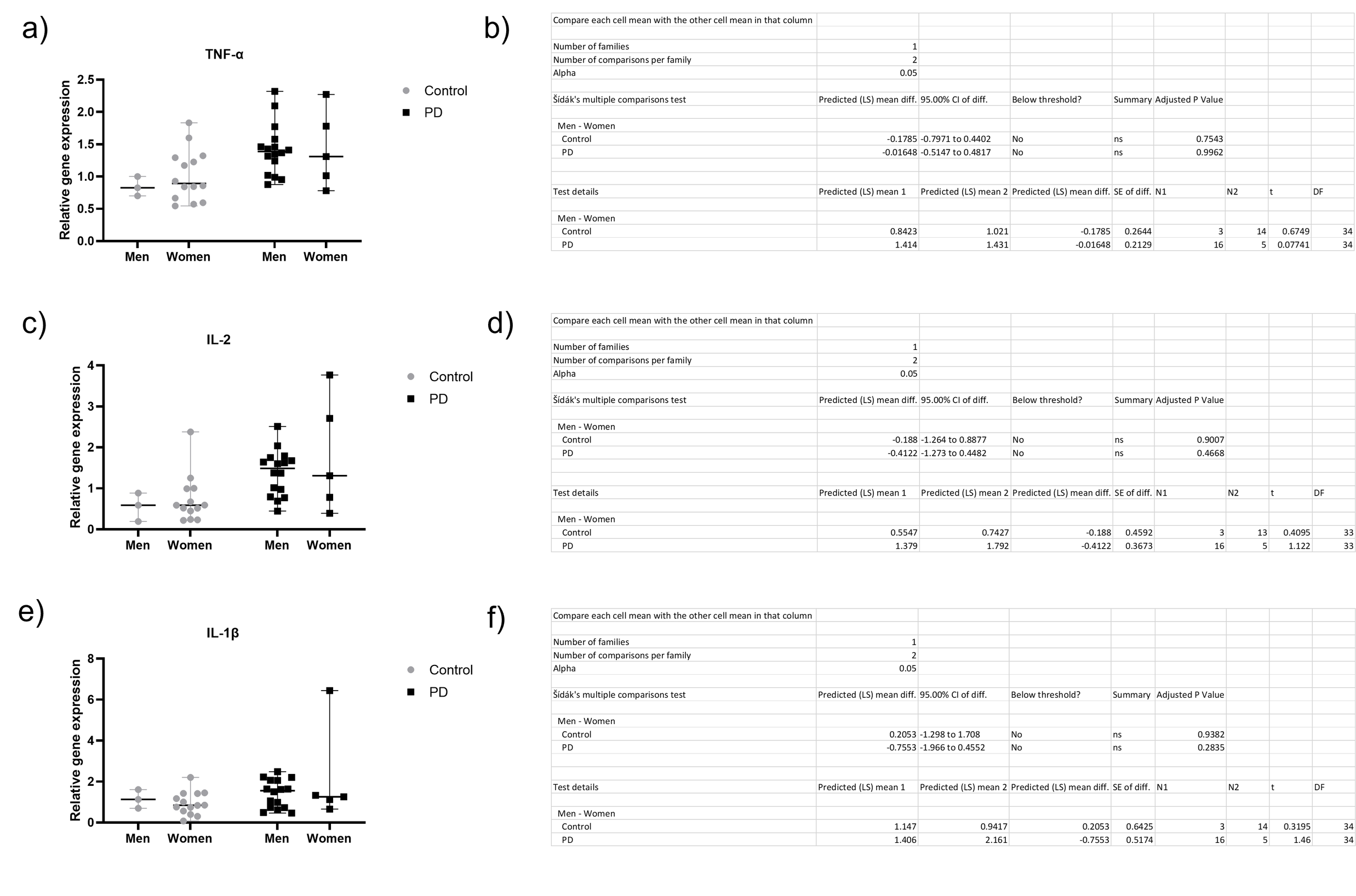

Supplement: S4 Fig — TNF-α (a, b), IL-2 (c, d), and IL-1β (e, f) gene expression did not differ between men and women in the healthy control and PD groups. Abbreviations: IL = interleukin, PD = Parkinson’s disease. Pearson’s correlation analysis, TNF-α = tumour necrosis factor alpha. P > 0.05. Two-Way ANOVA followed by Sidak’s multiple comparisons. (TIF) [file pone.0276564.s004.tif]
